# Self-esteem and subjective well-being revisited: The roles of personal, relational, and collective self-esteem

**DOI:** 10.1371/journal.pone.0183958

**Published:** 2017-08-25

**Authors:** Hongfei Du, Ronnel B. King, Peilian Chi

**Affiliations:** 1 Guangzhou University, Guangzhou, China; 2 The Education University of Hong Kong, Hong Kong, China; 3 University of Macau, Macau, China; Mälardalen University, SWEDEN

## Abstract

Previous studies have shown that self-esteem is an important predictor of subjective well-being. However, the majority of research has focused on self-esteem at the individual and the collective level, but has mostly ignored self-esteem at the relational level. According to social identity theory, individuals can maintain and enhance self-esteem through personal traits (personal self-esteem, PSE), relationships with significant others (relational self-esteem, RSE), and relationships with larger groups (collective self-esteem, CSE). The current research investigated whether RSE and CSE can predict subjective well-being beyond PSE among Chinese college students. With four cross-sectional studies and one longitudinal study (*N* = 847), we found that, when controlling for PSE, RSE was associated with greater life satisfaction, positive affect, meaning in life, happiness, and subjective vitality (Studies 1–5), but CSE was not (Studies 2–5). Implications are discussed.

## Introduction

Self-esteem has been a popular topic in psychology for many decades [1–5]. Researchers have found that self-esteem—defined as a person's sense of self-worth—is closely associated with well-being and a number of other adaptive outcomes. Despite the well-developed literature on self-esteem and its correlates, a key shortcoming is that majority of the studies have focused on self-esteem at the individual level (personal self-esteem, PSE) and more recently at the collective level (collective self-esteem, CSE). However, there is a lack of research on self-esteem at the relational level (relational self-esteem, RSE).

This is an important gap that needs to be filled given that individuals derive their sense of self-worth from multiple sources such as one's personal attributes, one's relationships with significant others, and one's membership in social groups [6–8]. By focusing mostly on PSE and to a lesser extent on CSE, not much is known about how the three types of self-esteem predict key outcomes especially when juxtaposed against each other.

One of the most important outcomes associated with self-esteem is subjective well-being [9–11]. It pertains to people's perceptions of their life quality which can involve cognitive evaluations (i.e., life satisfaction) and emotional reactions (i.e., positive affect) [12].

In an investigation of self-esteem and life satisfaction among 13,118 college students in 31 nations, Diener and Diener [9] found that self-esteem was moderately correlated with life satisfaction, though the strength of this association varied across cultures. Self-esteem was also found to be associated with other indicators of subjective well-being, such as positive and negative affect [13], meaning in life [14], and subjective vitality [15].

### Three types of self-esteem

The self can be conceptualized and measured at multiple levels. According to social identity theory, self-esteem can be derived from both the personal self and the social self [16]. The personal self refers to self-concept derived from one's unique traits and attributes that differentiate a person from others. Under the social self, we can further differentiate between the relational and the collective self [17, 18]. The relational self pertains to aspects of the self-concept that are rooted in interpersonal attachments and that consists of aspects shared with significant others (e.g., family, friends) and define one's roles in those relationships. The collective self refers to aspects of the self derived from membership from social groups (e.g., ethnic group). Numerous studies have shown that these three types of self-concept are empirically distinguishable from each other and have distinct effects on a wide range of psychological phenomena [8, 18].

Paralleling the three types of self-concept, it has also been found that people can evaluate their self-worth at the personal, relational, and collective level [6]. PSE is implicated when people derive their sense of self worth from their personal attributes such as their abilities and talents [1]. RSE pertains to self-worth derived from one's relationships with significant others such as family and friends [19, 20]. CSE pertains to self-worth derived from membership in larger social groups [21, 22].

### Self-esteem and subjective well-being

The literature on self-esteem suggests a strong link between self-esteem and subjective well-being. This linkage, however, varies with the type of self-esteem examined. For example, a large literature has demonstrated the positive role of PSE in promoting subjective well-being [9, 23, 24]. In addition, culture has been found to shape the strength of the association between PSE and subjective well-being. PSE was found to be correlated more strongly with life satisfaction in individualistic than in collectivistic societies [9, 25]. Individuals from individualistic cultures may put greater emphasis on their unique traits and personal attributes, which makes PSE more salient. In contrast, individuals in collectivist cultures may put greater emphasis on the relational and collective aspects of the self [26]. It is essential to clarify which type of self-esteem is beneficial for subjective well-being in collectivist cultures.

Crocker and her colleagues have extended self-esteem research from the individual level to collective level and investigated the link between CSE and subjective well-being [27]. According to social identity theory [28], the collective is an essential aspect of the self, and therefore evaluation of the collective self would contribute to subjective well-being. Indeed, they found that CSE was positively associated with well-being among White, Black, and Asian college students in the United States; however, when controlling for PSE, the relationship of CSE with well-being became nonsignificant for Whites, small for Blacks, and moderate to strong for Asians [27]. This suggests that culture may play a key role in determining the salience of the different types of self-esteem.

Bettencourt and Dorr [29] showed that CSE mediated the relation between allocentrism (defined as individual differences in collectivism) and subjective well-being among U.S. college students. In addition, the association between CSE and subjective well-being was also demonstrated among Chinese youth and adults, but CSE only explained a very small amount (i.e., 1%) of the variance in life satisfaction when controlling for PSE [30]. Furthermore, a recent study showed that the link between CSE and subjective well-being was mediated and accounted for by PSE [31]. These findings suggest that CSE is predictive of subjective well-being, but the prediction is rather weak when controlling for PSE among both Westerners and Asians.

Compared to the more extensive research on PSE and CSE, fewer studies examined the association between RSE and subjective well-being. The relational self is even more salient than the collective self, especially among collectivist cultures given their strong emphasis on the family [32]. Based on social identity theory [28], if the relational self is an important aspect of one’s selfhood, then one’s evaluation of therelational self should be related to subjective well-being. Unfortunately, most of the existing evidence is indirect at best. Although there are very few studies on RSE per se, existing research on social relationships has accumulated a strong body of evidence pertaining to the importance of interpersonal attachments in enhancing well-being [33]. For example, studies have shown that individuals who felt more related and connected to significant others such as their family and friends were more likely to exhibit optimal functioning [34, 35]. Numerous studies have also shown that individuals who have a secure attachment with their parents had higher levels of psychological well-being [36, 37].

Kwan and her colleagues [38] found that PSE and relationship harmony had an additive effect on predicting life satisfaction. Relationship harmony pertains to achieved mutuality and the degree of harmony for relationships with significant others. This finding on relationship harmony indicates that close relationships may boost subjective well-being. However, their study did not examine whether individuals can maintain self-esteem through close relationships to further improve their subjective well-being.

One of the few studies that directly examined RSE found that RSE was correlated with multiple indicators of well-being (e.g., positive affect, meaning in life, depression) and these associations held even when controlling for PSE [39]. However, a shortcoming of this study was that it was conducted with a sample of vulnerable children (i.e., children affected by parental HIV) so that the findings may not necessarily be generalizable to other populations. In addition, although PSE was partialled out, this study did not consider the potential explanatory power of CSE in accounting for variance in subjective well-being. The predictive power of RSE might have been overestimated in the absence of CSE.

The present study aimed to fill these gaps and investigated the roles of PSE, RSE, and CSE in promoting subjective well-being. The findings will help us gain a comprehensive understanding of the relationship between self-esteem and subjective well-being. If RSE and CSE do have effects on subject well-being, independent of PSE, then subjective well-being research may need to be expanded to include multiple aspects of the self.

### Overview of the present studies

The aim of the current research was to investigate the roles of PSE, RSE, and CSE in subjective well-being. We examined whether PSE, RSE, and CSE would have additive, positive effects on subjective well-being. Our studies were conducted with Chinese samples. Chinese collectivistic culture emphasizes the pursuit of self-esteem in the context of close relationships [26, 40]. Moreover, research among Chinese populations has revealed that individuals are more likely to be motivated by the relational self than the collective self [7]. We therefore hypothesized that in addition to PSE, RSE would be a significant predictor of subjective well-being, but CSE would not be associated with subjective well-being.

We conducted five studies to test our hypotheses about the relations between self-esteem and subjective well-being. Study 1 aimed to replicate previous research [39] by examining whether RSE can account for additional variance in well-being after accounting for PSE. We conducted a survey to examine intercorrelations among PSE, RSE, and three indicators of subjective well-being (i.e., life satisfaction, positive affect, and meaning in life). Study 2 tested CSE in addition to PSE and RSE as predictors for two indicators of subjective well-being (i.e., meaning in life, subjective vitality). Study 3 examined PSE, RSE, and CSE as predictors for two other indicators of subjective well-being (i.e., life satisfaction, happiness). Moreover, we tested whether the effects of self-esteem on subjective well-being held when controlling for independent and interdependent self-construal, because extant research suggests that self-construal, as a closely related construct to self-esteem, contributes to better well-being [38, 41]. Study 4 aimed to replicate the findings in Study 3 by showing that self-esteem predicts subjective well-being (i.e., life satisfaction, positive affect) when controlling for independent and interdependent self-construal. Study 5 was a longitudinal study that followed participants for a month and examined whether PSE, RSE, and CSE would predict long-term life satisfaction even when controlling for prior levels of life satisfaction. Across the five studies, we included multiple indicators of subjective well-being, including life satisfaction, positive affect, meaning in life, subjective vitality, and happiness. If the five studies using different indicators of well-being can provide convergent evidence, we would have stronger confidence in the effects of the three types of self-esteem on subjective well-being.

Considering three types of self-esteem were associated with each other and used as predictors in the five studies, we examined whether there would be a multicollinearity problem by using an index of tolerance statistics. A tolerance value of less than 0.20 indicates a potential multicollinearity problem [42]. The tolerance statistics were higher than .28 in all five studies, suggesting that multicollinearity is not an issue in the current analyses.

## Study 1

The goal of Study 1 was to examine associations of PSE and RSE with various indicators of well-being such as life satisfaction, positive affect, and meaning in life. We predicted that both PSE and RSE would be positively associated with life satisfaction, positive affect, and meaning in life. More importantly, RSE would predict the three outcomes, even after accounting for the variance associated with PSE.

### Method

The Ethics Committee in Department of Psychology at University of Macau has approved this study (approval number: 2015–23).

#### Participants

The sample included 179 college students in Macau, China (94 women, with average age 18.99, *SD* = 1.214) who participated in the study in exchange for course credit.

#### Materials and procedures

Participants completed the survey online. After providing informed consent, participants completed a survey containing measures on the constructs.

As a measure of PSE, participants completed the 10-item Rosenberg Self-Esteem Scale [1] which measured global sense of personal worth (e.g., “I feel that I have a number of good qualities”). Participants answered the items on a 4-point Likert scale ranging from 1 (strongly disagree) to 4 (strongly agree). Items were averaged, with a higher score indicating higher PSE. (α = .72)

RSE was assessed with the 8-item Relational Self-Esteem Scale [39] which measured a sense of self-worth pursued through relationships with significant others (e.g., “I am proud of my family”). Participants answered the items on a 4-point Likert Scale ranging from 1 (strongly disagree) to 4 (strongly agree). Items were averaged, with a higher score indicating higher RSE (α = .85).

Life satisfaction was measured with the 5-item Life Satisfaction Scale [43] which measured overall satisfaction with one’s own life (e.g., “I am satisfied with my life”). Participants answered the items on a 7-point Likert scale ranging from 1 (*strongly disagree*) to 7 (*strongly agree*). Items were averaged, with a higher score indicating higher life satisfaction (α = .84).

Positive affect was measured with the 10-item positive affect subscale of the Positive and Negative Affect Schedule [44] which measured 10 different positive moods (e.g., “enthusiastic”). Participants answered the items on a 5-point scale ranging from 1 (*slightly or not at all*) to 5 (*very much*). Items were averaged, with a higher score indicating more positive affect (α = .90).

Meaning in life was measured with the 5-item presence of meaning subscale of the Meaning in Life Questionnaire which measured the presence of meaning or purpose in a person’s life (e.g., “I understand my life’s meaning”) [14]. Participants answered the items on a 5-point scale ranging from 1 (*strongly disagree*) to 7 (*strongly agree*). Items were averaged, with a higher score indicating more meaning in life (α = .78).

All instruments were administered in Chinese. The Chinese versions of the instruments have shown good psychometric properties in previous studies [40, 45, 46]. Cronbach’s alphas of the instruments in the current study also indicate acceptable reliabilities.

### Results and discussion

The descriptive statistics and correlations between the predictors and subjective well-being in Study 1 can be seen in Table 1 (Data of all five studies are available at https://osf.io/6dcgs/). We conducted two-step hierarchical regression analyses examining the contribution of PSE and RSE to explaining variance in life satisfaction, positive affect, and meaning in life, respectively. We introduced PSE as a predictor in the first step and introduced RSE as a predictor in the second step, allowing us to examine the unique contribution of each type of self-esteem.

**Table 1:** Descriptive statistics and bivariate correlations (Study 1).

Variables	*M*	*SD*	1	2	3	4	5
1. Personal Self-Esteem	2.77	0.39	1	.54***	.34***	-.03	.37***
2. Relational Self-Esteem	3.01	0.40		1	.47***	.23**	.39***
3. Life Satisfaction	4.55	0.93			1	.21**	.51***
4. Positive Affect	2.96	0.57				1	.27***
5. Meaning in Life	4.41	0.89					1

Note.

**p < .01

***p < .001.

As can be seen in Table 2, findings indicated that PSE was significantly associated with more life satisfaction and meaning in life, but not significantly associated with positive affect. That is, participants with high PSE felt greater life satisfaction and purpose in life, but did not show elevated positive moods. When RSE was entered in the regression, PSE was not a significant predictor of life satisfaction anymore. In contrast, RSE significantly predicted life satisfaction, suggesting that RSE may contribute to satisfaction with one’s own life more than PSE. RSE was also a significant predictor of positive affect and meaning in life, suggesting that people with high RSE experienced more positive affect and purpose in life. Considering the null correlation between PSE and positive affect, PSE acted as a suppressor variable in the relationship between RSE and positive affect [47].

**Table 2:** Standardized regression coefficients for the prediction of life satisfaction and positive affect from personal Self-Esteem and relational Self-Esteem (Study 1).

	Step 1		Step 2	
Effects	β	SE	β	SE
*Life Satisfaction*				
Personal Self-Esteem	0.34***	0.17	0.13	0.19
Relational Self-Esteem			0.40***	0.18
R^2^_Δ_	0.12***		0.21***	
*Positive Affect*				
Personal Self-Esteem	-0.03	0.11	-0.21*	0.13
Relational Self-Esteem			0.34***	0.12
R^2^_Δ_	0.001		0.08***	
*Meaning in Life*				
Personal Self-Esteem	0.37***	0.16	0.23**	0.185
Relational Self-Esteem			0.27***	0.179
R^2^_Δ_	0.14***		0.05**	

Note.

*p < .05

**p < .01

***p < .001.

Overall, the findings were in line with our predictions showing that RSE was associated with life satisfaction, positive affect, and meaning in life. In contrast, PSE was not significantly associated with positive affect, which was different from previous findings [13]. These findings suggest that RSE provides additional explanatory power in predicting subjective well-being. Study 2 aimed to replicate these findings and also included CSE as a predictor in addition to PSE and RSE.

## Study 2

While Study 1 documented evidence that RSE predicted additional variance in well-being outcomes even after controlling for PSE, it did not include CSE as a crucial predictor. Thus, it is not known whether the positive association between RSE and various well-being outcomes will hold once CSE and PSE are both included in the model.

The goal of Study 2 therefore was to further examine the link between RSE and subjective well-being in the presence of CSE. Specifically, we included CSE as an additional predictor and tested whether CSE would provide an additive prediction for subjective well-being beyond PSE and RSE. We also examined whether the findings of Study 1 that RSE was a significant predictor of subjective well-being would be replicated.

Meaning in life was used as an indicator of well-being as in Study 1; moreover, we added another commonly used indicator: subjective vitality which refers to one’s conscious experience of possessing energy and vitality [15].

### Method

#### Participants

A sample of 151 college students in Macau, China (107 women, with average age 19.47, *SD* = 1.42) who participated in the study in exchange for course credit.

#### Materials and procedures

Participants completed the survey in separate cubicles in the laboratory. After providing informed consent, participants responded to the measures on three types of self-esteem, subjective vitality, and meaning in life. Instruments used to measure PSE (α = .81), RSE (α = .84), and meaning in life (α = .86) were the same as those used in Study 1.

CSE was measured with the 16-item Collective Self-Esteem Scale [22], which assessed one’s worth in relation to general social groups. We operationalized CSE as self-worth derived from participants’ identity as Macau residents and adapted all the items to assess their CSE as Macau residents (e.g., “I feel good about being a Macau resident”). This is in line with previous research which has shown that individuals can derive self-esteem from their ethnic identity [48]. Participants answered the items on a 4-point Likert scale ranging from 1 (*strongly disagree*) to 4 (*strongly agree*). Items were averaged, with a higher score indicating higher CSE (α = .74).

Individuals’ collective self-esteem could be dependent on their identification with their ethnic group, and group identification has been found to be associated with well-being [49]. Therefore, in this study, we also assessed participants’ identification with their ethnic group using two items: “Do you identify yourself as a Macau resident?” and “Would you like to be seen as a Macau resident?” Participants answered the items on a 5-point Likert scale ranging from 1 (*strongly disagree*) to 5 (*strongly agree*) (α = .92).

Subjective vitality was assessed with the 7-item Subjective Vitality Scale [15], which measured a sense of aliveness and energy. Participants answered the items (e.g., “I feel alive and vital”) on a 7-point Likert scale ranging from 1 (*not at all true*) to 7 (*very true*). Items were averaged, with a higher score indicating greater feelings of energy and aliveness (α = .86).

### Results and discussion

A preliminary analysis showed that participants had a strong identification with Macau (*M* = 4.00, *SD* = .78). Identification was significantly associated with CSE (*r* = .19, *p* = .02). To minimize the impact of identification on the relationship between CSE and subjective well-being, we excluded 6 participants with low identification (i.e., participants who indicated that they didn’t identify themselves as Macau residents; identification values < 3). Therefore, the sample of 145 participants left in the further analysis represented a group of participants with strong identification with Macau.

The descriptive statistics and correlations between the predictors and subjective well-being can be seen in Table 3. To test our predictions, we conducted two hierarchical regression analyses to examine the associations of three types of self-esteem with meaning in life and subjective vitality, respectively. In both regression analyses, again, PSE was entered as a predictor in the first step, and RSE and CSE were introduced in the second step.

**Table 3:** Descriptive statistics and bivariate correlations (Study 2).

Variables	*M*	*SD*	1	2	3	4	5
1. Personal Self-Esteem	2.79	0.37	1	.54***	.36***	.57***	.40***
2. Relational Self-Esteem	3.07	0.39		1	.34***	.58***	.42***
3. Collective Self-Esteem	2.83	0.24			1	.34***	.31***
4. Subjective Vitality	4.82	0.78				1	.45***
5. Meaning in Life	4.82	0.78					1

Note.

***p < .001.

As can be seen in Table 4, PSE was positively associated with both meaning in life and subjective vitality, and these effects were still significant when RSE and CSE were introduced in the regression equation. RSE was also positively associated with both meaning in life and subjective vitality. In contrast, CSE showed only a marginally significant association with meaning in life and no significant association with subjective vitality.

**Table 4:** Standardized regression coefficients for the prediction of subjective vitality and meaning in life from personal Self-Esteem, relational Self-Esteem, and collective Self-Esteem (Study 2).

	Step 1		Step 2	
Effects	β	SE	β	SE
*Subjective Vitality*				
Personal Self-Esteem	0.57***	0.15	0.34***	0.16
Relational Self-Esteem			0.36***	0.15
Collective Self-Esteem			0.1	0.22
R^2^_Δ_	0.33***		0.11***	
*Meaning in Life*				
Personal Self-Esteem	0.40***	0.20	0.21*	0.24
Relational Self-Esteem			0.26**	0.22
Collective Self-Esteem			0.14^+^	0.32
R^2^_Δ_	0.16***		0.08**	

Note.

^+^*p* = .08

*p < .05

**p < .01

***p < .001.

These results replicated the findings of Study 1 which found that RSE was a significant predictor of subjective well-being, even when controlling for PSE. In addition, CSE was found to be a weaker predictor relative to PSE and RSE. These findings are consistent with our hypotheses.

## Study 3

The goal of Study 3 was to replicate the findings in Study 2 that RSE was a significant predictor of subjective well-being when using a different outcome variable and when controlling for self-construal. In Study 3, we used another important indicator of subjective well-being, happiness, together with life satisfaction. Based on the results of Study 2, we predicted that both PSE and RSE would be associated with more life satisfaction and greater happiness but that CSE would be a weaker predictor.

In Study 3, we wanted to investigate whether the relations between self-esteem and subjective well-being would hold when controlling for self-construal. Previous studies have shown that independent and interdependent self-construals were significant predictors of subjective well-being [38, 41]. It is plausible that not all three types of self-esteem would predict well-being after controlling for self-construal. We therefore included independent and interdependent self-construals as covariates and tested whether PSE, RSE, and CSE would still account for additional variance in well-being. If PSE, RSE, and CSE could demonstrate incremental predictive validity beyond the effects of self-construal then we would have greater confidence in the utility of the self-esteem constructs in understanding well-being.

### Method

#### Participants

A sample of 118 college students in Macau, China (69 women, with average age 19.36, *SD* = 1.29) who participated in the study in exchange for course credit.

#### Materials and procedure

Participants completed the survey in separate cubicles in the laboratory. After providing informed consent, participants responded to a questionnaire measuring the three types of self-esteem, life satisfaction, happiness, and independent and interdependent self-construal. The measures of PSE (α = .79), RSE (α = .82), and life satisfaction (α = .88) were the same as those used in Study 1. The measure of CSE (α = .82) was the same as that used in Study 2.

Happiness was assessed with the 4-item Subjective Happiness Scale [50], which measured global subjective happiness. Participants answered the items (e.g., “Compared to most of my peers, I consider myself less/more happy”) on a 7-point Likert scale ranging from 1 (*very unhappy*) to 7 (*very happy*). Items were averaged, with a higher score indicating greater happiness (α = .75).

Independent and interdependent self-construals were measured with the 16-item Individualism-Collectivism Scale [51], which measured one’s individualistic (e.g., "I'd rather depend on myself than others.") and collectivistic orientations (e.g., "I feel good when I cooperate with others."). Participants answered the items on a 4-point Likert scale ranging from 1 (strongly disagree) to 4 (strongly agree). Items were averaged for independent self-construal (α = .71) and interdependent self-construal (α = .71), respectively, with a higher score indicating stronger self-construal.

### Results and discussion

The descriptive statistics and correlations for Study 3 can be seen in Table 5. To test our predictions, we conducted two hierarchical regression analyses to examine the associations of the three types of self-esteem with life satisfaction and happiness, respectively. In both regression analyses, independent and interdependent self-construals were entered as predictors in the first step; PSE was entered as a predictor in the second step; and RSE and CSE were introduced in the third step.

**Table 5:** Descriptive statistics and bivariate correlations (Study 3).

Variables	*M*	*SD*	1	2	3	4	5	6	7
1. Personal Self-Esteem	2.76	0.34	1	.57***	.41***	.66***	.47***	.24**	.22*
2. Relational Self-Esteem	2.98	0.35		1	.38***	.62***	.41***	.14	.31**
3. Collective Self-Esteem	2.75	0.36			1	.35***	.33***	.05	.30**
4. Life Satisfaction	4.70	1.16				1	.64***	.00	.19*
5. Happiness	4.46	0.94					1	-.07	.18^+^
6. Independent self-construal	3.47	0.53						1	.11
7. Interdependent self-construal	3.69	0.45							1

Note.

^+^*p* = .06

*p < .05

**p < .01

***p < .001.

As can be seen in Table 6, PSE was positively associated with both life satisfaction and happiness, and these effects were still significant when RSE and CSE were introduced in the regression model. RSE was also positively associated with both life satisfaction and happiness. In contrast, CSE showed no significant association with either life satisfaction or happiness.

**Table 6:** Standardized regression coefficients for the prediction of life satisfaction and happiness from personal Self-Esteem, relational Self-Esteem, and collective Self-Esteem (Study 3).

	Step 1		Step 2		Step 3	
Effects	β	SE	β	SE	β	SE
*Life Satisfaction*						
Independent self-construal	-0.02	0.20	-0.17*	0.16	-0.16*	0.14
Interdependent self-construal	0.19*	0.24	0.06	0.18	-0.02	0.18
Personal Self-Esteem			0.69***	0.25	0.47***	0.28
Relational Self-Esteem					0.36***	0.26
Collective Self-Esteem					0.03	0.23
R^2^_Δ_	0.04		0.43***		0.09***	
*Happiness*						
Independent self-construal	-0.09	0.16	-0.2	0.15	-0.19*	0.15
Interdependent self-construal	0.19*	0.19	0.09	0.17	0.03	0.18
Personal Self-Esteem			0.49***	0.23	0.36**	0.28
Relational Self-Esteem					0.18^+^	0.27
Collective Self-Esteem					0.12	0.24
R^2^_Δ_	0.04		0.22***		0.04^+^	

Note.

^+^*p* < .10

*p < .05

**p < .01

***p < .001.

Overall, the findings replicated the observed associations of the three types of self-esteem with subjective well-being in Study 2. Results of Study 3 indicated that people with high PSE and RSE reported more life satisfaction, but this effect was not observed for CSE. Study 3 also extended Study 2 by demonstrating the predictive effect of PSE and RSE on happiness. CSE was not a significant predictor of happiness. In addition, the predictions of PSE and RSE held even when controlling for independent and interdependent self-construals.

## Study 4

The goal of Study 4 was to extend Studies 1–3. In Studies 2–3, CSE was operationalized as self-worth derived from participants’ identity as Macau residents. In Study 4, we used *nationality* as a different operationalization of CSE. Nationality is one of the most common group identities identified in previous research [21]. Hence, we operationalized CSE as self-worth derived from participants’ identity as Chinese in Study 4. In addition, we included independent and interdependent self-construals as covariates, similar to what we did in Study 3. In Study 4, we adopted life satisfaction and positive affect as indicators of subjective well-being, using the same measures as those used in Study 1.

### Method

#### Participants

A sample of 297 college students in Macau, China (167 women, with average age 18.94, *SD* = 1.21) who participated in the study in exchange for course credit.

#### Materials and procedure

Participants completed the survey online. After providing informed consent, participants completed a survey containing measures on the constructs. The measures on PSE (α = .85), RSE (α = .81), life satisfaction (α = .87), and positive affect (α = .87) were the same as those used in Study 1. The measures of independent self-construal (α = .68) and interdependent self-construal (α = .69) were the same as those used in Study 4.

The measure of CSE was the same as that used in Study 3, except that we operationalized CSE as participants’ self-esteem in relation to their Chinese identity. Specifically, we adapted all the items to assess their CSE as a Chinese (e.g., “I feel good about being a Chinese”) (α = .88).

### Results and discussion

The descriptive statistics and correlations for Study 4 can be seen in Table 7. We conducted two hierarchical regression analyses to examine the associations of three types of self-esteem with life satisfaction and positive affect, respectively. The analytic methods were the same as Study 3 (see Table 8).

**Table 7:** Descriptive statistics and bivariate correlations (Study 4).

Variables	*M*	*SD*	1	2	3	4	5	6	7
1. Personal Self-Esteem	2.83	0.45	1	.62***	.39***	.48***	.52***	0.01	.21***
2. Relational Self-Esteem	2.97	0.37		1	.42***	.44***	.54***	.12*	.43***
3. Collective Self-Esteem	2.57	0.40			1	.26***	.30***	-0.03	.19**
4. Life Satisfaction	4.70	1.04				1	.39***	-0.03	.34***
5. Positive Affect	3.14	0.61					1	.29***	.25***
6. Independent self-construal	3.46	0.50						1	.16**
7. Interdependent self-construal	3.65	0.46							1

Note.

*p < .05

**p < .01

***p < .001.

**Table 8:** Standardized regression coefficients for the prediction of life satisfaction and positive affect from personal Self-Esteem, relational Self-Esteem, and collective Self-Esteem (Study 4).

	Step 1		Step 2		Step 3	
Effects	β	SE	β	SE	β	SE
*Life Satisfaction*						
Independent self-construal	-0.09	0.12	-0.07	0.1	-0.08	0.1
Interdependent self-construal	0.35***	0.13	0.26***	0.12	0.22***	0.12
Personal Self-Esteem			0.43***	0.13	0.35***	0.16
Relational Self-Esteem					0.14*	0.19
Collective Self-Esteem					0.02	0.14
R^2^_Δ_	0.12***		0.18***		0.01^+^	
*Positive Affect*						
Independent self-construal	0.26***	0.07	0.27***	0.06	0.26***	0.06
Interdependent self-construal	0.21***	0.07	0.10*	0.07	0.02	0.07
Personal Self-Esteem			0.48***	0.07	0.30***	0.09
Relational Self-Esteem					0.30***	0.11
Collective Self-Esteem					0.06	0.08
R^2^_Δ_	0.13***		0.22***		0.06***	

Note.

^+^*p* < .10

*p ≤ .05

***p < .001.

First, we found that PSE was associated with both life satisfaction and positive affect. PSE was still a significant predictor after RSE and CSE were introduced in the regression. RSE was also a significant predictor for both life satisfaction and positive affect. In contrast, CSE was not a significant predictor of either life satisfaction or positive affect.

These findings conceptually replicated and extended the findings of Studies 1–3: Participants with high RSE reported greater life satisfaction and more positive affect. PSE was still a significant predictor of both life satisfaction and positive affect. Consistent with the findings of Studies 2–3, CSE did not emerge as a predictor of either life satisfaction or positive affect.

## Study 5

Study 5 aimed to further examine the link between three types of self-esteem and well-being while addressing methodological limitations imposed by the cross-sectional designs of Studies 1–4. For this purpose, we conducted a 1-month longitudinal study and surveyed students’ self-esteem and well-being at two time points. The study investigated whether, when controlling for baseline differences, changes in the types of self-esteem would predict changes in subsequent well-being.

An important strength of Study 5 is that we measured the different types of self-esteem and the outcome variable across the two time points. When the prior level of an outcome (e.g., well-being) is not statistically accounted for, the evidence of prospective effects can be confounded by the concurrent relations between the constructs. Estimates of prospective effects might be somewhat misleading [52]. Therefore, in this study we also controlled for the effects of Time 1 well-being on Time 2 well-being.

We hypothesized that higher PSE and RSE over time would be associated with better well-being, whereas CSE would not be a significant predictor. In this study, subjective well-being was operationalized as life satisfaction.

### Method

#### Participants

Participants were 111 college students in Macau, China (70 women, 36 men, and 5 didn’t report gender) with average age of 19.26 years (*SD* = 1.15). Students participated in the study in exchange for course credit.

#### Design and procedure

The study used a longitudinal design. Students who agreed to participate in the study completed the Time 1 survey online. They provided their email address so that they can be contacted to participate at Time 2. Among the 111 participants, 7 participants were excluded from further analysis: 5 participants did not complete the survey and 2 participants completed the Time 1 survey less than 3 minutes (They also responded to all items—both positively and negatively worded items—with the same choice in the Likert scale). Hence, these 7 participants were not contacted to participate in the Time 2 survey, leaving 104 potential participants for the Time 2 survey. The Time 2 survey (*N* = 82) was collected 1 month later. Among the 82 participants, 8 participants were excluded from further analysis: 3 participants did not complete the survey and 5 participants completed the Time 2 survey less than 3 minutes (They also responded to all items with the same choice in the Likert scale). Hence, the final sample was 74 participants at the Time 2 point.

#### Materials

Both the Time 1 and Time 2 surveys included measures on PSE, RSE, CSE, and life satisfaction. The measures of PSE, RSE, CSE, and life satisfaction were the same as those used in Study 3. All the measures showed acceptable reliabilities with Cronbach’s alphas > .74, as shown in Table 9.

**Table 9:** Descriptive statistics and bivariate correlations (Study 5).

Variables	*M*	*SD*	Cronbach’s alpha	T1 PSE	T1 RSE	T1 CSE	T1 LS	T2 PSE	T2 RSE	T2 CSE	T2 LS
T1 PSE	2.75	0.38	0.84	1	.69***	.41***	.64***	.75***	.50***	0.21	.55***
T1 RSE	2.99	0.39	0.85		1	.29**	.67***	.60***	.70***	0.18	.52***
T1 CSE	2.75	0.24	0.74			1	.25*	.31**	.28*	.74***	.26*
T1 LS	4.62	1.14	0.91				1	.55***	.54***	0.15	.82***
T2 PSE	2.72	0.35	0.87					1	.59***	.27*	.50***
T2 RSE	3.03	0.34	0.87						1	.33**	.53***
T2 CSE	2.74	0.28	0.82							1	0.21
T2 LS	4.59	1.10	0.91								1

Note.

*p < .05

**p < .01

***p < .001.

PSE = personal self-esteem; RSE = relational self-esteem; CSE = collective self-esteem; LS = life satisfaction.

### Results and discussion

We conducted a regression analysis predicting Time 2 life satisfaction while controlling for Time 1 life satisfaction. We included the three types of self-esteem at both Time 1 and Time 2 as predictors. Table 10 shows the longitudinal relationships among the variables.

**Table 10:** Standardized regression coefficients for the prediction of life satisfaction from personal Self-Esteem, relational Self-Esteem, and collective Self-Esteem at two time points (Study 5).

	Step 1		Step 2		Step 3	
Effects	β	SE	β	SE	β	SE
Block 1						
T1 Life Satisfaction	0.88***	0.09	0.88***	0.09	0.87***	0.09
T1 Personal Self-Esteem	0.07	0.28	0.00	0.33	0.07	0.34
T1 Relational Self-Esteem	-0.17	0.28	-0.18	0.28	-0.30*	0.31
T1 Collective Self-Esteem	0.04	0.35	0.04	0.35	-0.04	0.50
Block 2						
T2 Personal Self-Esteem			0.12	0.32	0.03	0.34
Block 3						
T2 Relational Self-Esteem					0.20^+^	0.33
T2 Collective Self-Esteem					0.08	0.40
R^2^_Δ_	0.69***		0.01		0.02^+^	

Note.

^+^*p* < .08

*p ≤ .05

***p < .001.

We regressed T2 life satisfaction on T1 life satisfaction, T1 PSE, T1 RSE and T1 CSE which were all entered in Block 1. To control for the predictive effect of T2 PSE, we included T2 PSE in Block 2. Lastly, we included T2 RSE and T2 CSE in Block 3. As shown in Step 1, the T1 variables predicted T2 life satisfaction, although only T1 life satisfaction was a significant predictor. This pattern did not change when T2 PSE was added as a predictor in Step 2. In Step 3, T1 life satisfaction was still significantly associated with T2 life satisfaction. More importantly, T2 RSE emerged as a significant predictor for T2 life satisfaction, though the effect was marginally significant (*p* = .057). In contrast, T2 PSE and T2 CSE were not significant predictors. In addition, we also observed that T1 RSE became a significant predictor of T2 life satisfaction. Considering the positive bivariate correlation between T1 RSE and T2 life satisfaction (*r* = .54), the negative association of T1 RSE with T2 life satisfaction in the regression equation could be regarded as a suppression effect.

Overall, the findings of Study 5 replicated what we found in Studies 1–4: participants high in RSE reported greater life satisfaction, whereas CSE was a nonsignificant predictor of life satisfaction. In contrast, PSE was not a significant predictor of T2 life satisfaction after controlling for baseline life satisfaction. Although this finding was surprising, it was consistent with what we found in Study 1 wherein PSE was not associated with life satisfaction when controlling for RSE.

## General discussion

In recent years, researchers have distinguished three unique self-aspects and three types of self-esteem [7, 8, 18, 32]. But are all three types of self-esteem associated with subjective well-being? The five studies reported here offer a tentative answer: Both PSE and RSE are positive predictors of subjective well-being, and it seems that RSE is more salient compared to PSE at least in the collectivist Chinese context. CSE was only weakly associated with subjective well-being. Specifically, when controlling for PSE, RSE was positively associated with life satisfaction (Studies 1, 3 and 4), positive affect (Studies 1 and 4), meaning in life (Studies 1 and 2), and happiness (Study 3). Moreover, RSE was associated with greater life satisfaction over a 1-month period (Study 5). In contrast, CSE was not a significant predictor of subjective well-being in any of the five studies.

The findings of RSE replicate previous studies [39, 53] showing that people high in RSE tend to have better subjective well-being. The current study extends previous research in three ways. First, multiple indicators of subjective well-being were utilized including life satisfaction, positive affect, meaning in life, subjective vitality, and happiness. RSE was found to be positively associated with different aspects of subjective well-being, suggesting that the pursuit of self-worth through one's relationships with significant others might make people feel more positive and happy and be more satisfied with their lives. Second, the relationship between RSE and subjective well-being held even when controlling for several covariates including PSE, CSE, and independent and interdependent self-construal. This indicates the unique role of RSE in predicting well-being. Third, Study 5 adopted a longitudinal design, which addressed the methodological limitations of previous cross-sectional studies. We showed that individuals may optimize their well-being over time when they are able to maintain high levels of self-esteem through their relationships with significant others.

The positive association of PSE with subjective well-being is in line with previous findings [9, 13–15, 54] showing that people high in self-esteem tend to report stronger life satisfaction, positive affect, meaning in life, and subjective vitality. Interestingly, in our study, PSE was not associated with life satisfaction when controlling for RSE and CSE; instead, RSE emerged as a stronger predictor (Studies 1, 2, and 5). These findings were consistent with the cross-cultural literature which shows that among East Asians, interdependent self-construal is more salient compared to the independent self-construal [26] as well as previous research [38] showing that the predictive effect of PSE on well-being among East Asians was not as strong as the effect among Westerners. The findings of the current study with regard to the primacy of RSE also corroborate previous experimental research which showed that East Asians mainly relied on RSE (but not PSE) to buffer death-related anxiety, but Westerners mainly counted on PSE (but not RSE) to reduce anxiety [40]. We suggest that RSE may be a stronger predictor of adaptive outcomes compared to PSE among more collectivist East Asians at least.

In contrast to PSE and RSE, CSE was not associated with subjective well-being across the four studies (perhaps an exception is that CSE was marginally associated with meaning in life in Study 2). This result appears surprising because previous findings showed that CSE contributed to subjective well-being, even after controlling for PSE [27, 31]. We conjecture that the non-significant association between CSE and well-being might be due to the lower priority allocated to the collective self (even among East Asians). Recently, researchers have found that both the individual self and the relational self have greater motivational primacy compared to the collective self [7]. If individuals do not consider the collective self as an important aspect of self-identity, it is reasonable to observe that CSE is not systematically associated to life satisfaction. These findings suggest that CSE may not be as relevant as PSE and RSE in understanding subjective well-being.

Considering that culture profoundly affects how people evaluate themselves [26], we suspect that among the three types of self-esteem, PSE could be a reliable predictor of well-being among Westerners and RSE might also be associated with well-being. However, CSE might have a very limited predictive effect on subjective well-being. Future studies need to examine the associations of the three types of self-esteem with subjective well-being in individualistic societies.

Although we observed a consistent pattern with RSE across five studies including a longitudinal survey, the conclusions should be interpreted with caution because our data were correlational and do not indicate causal relations. While it is plausible that RSE contributes to subjective well-being, we cannot rule out that subjective well-being leads to better self-esteem. To answer these questions, future research can utilize both longitudinal and experimental studies to establish the causal relationship between self-esteem and subjective well-being. In addition, our studies exclusively relied on college students’ self-reports of RSE and CSE. Future research might consider gathering information about individuals’ self-esteem from other sources, such as their close others, classmates, and teachers. Last, multiple indicators of well-being adopted in each study were correlated with each other so that the prediction of self-esteem on one indicator (e.g., life satisfaction) may be partially due to the association of self-esteem with another indicator of well-being (e.g., happiness). Future studies are needed to replicate the current findings by controlling for shared variance among multiple indicators of well-being (e.g., using structural equation modeling).

In spite of these limitations, the five studies presented here consistently demonstrated that self-esteem at the relational level (but not the collective level) is robustly related to subjective well-being. These findings contribute substantially to current theorizing on self-esteem and may be useful in developing interventions aimed at optimizing individuals' well-being.

## References

[1] Rosenberg M. Society and the adolescent self-image Princeton, NJ: Princeton University Press 1965.

[2] Leary MR, Baumeister RF. The Nature and Function of Self-Esteem: Sociometer Theory In: Zanna MP, editor. Advances in Experimental Social Psychology. 32 New York: Academic Press; 2000 p. 1–62.

[3] Pyszczynski T, Greenberg J, Solomon S, Arndt J, Schimel J. Why do people need self-esteem: A theoretical and empirical review. Psychological Bulletin. 2004;130:435–68. doi: 10.1037/0033-2909.130.3.435 15122930 10.1037/0033-2909.130.3.435

[4] Crocker J, Park LE. The Costly Pursuit of Self-Esteem. Psychological Bulletin. 2004;130:392–414. doi: 10.1037/0033-2909.130.3.392 15122925 10.1037/0033-2909.130.3.392

[5] Greenwald AG, Banaji MR. Implicit social cognition: Attitudes, self-esteem, and stereotypes. Psychol Rev. 1995;102:4–27. 7878162 10.1037/0033-295x.102.1.4

[6] Breckler SJ, Greenwald AG. Motivational facets of the self In: Sorrentino RM, Higgins ET, editors. Handbook of motivation and cognition. 1 New York: Guilford Press; 1986 p. 145–64.

[7] Gaertner L, Sedikides C, Luke M, O'Mara EM, Iuzzini J, Jackson LE, et al A motivational hierarchy within: Primacy of the individual self, relational self, or collective self? Journal of Experimental Social Psychology. 2012;48(5):997–1013.

[8] Sedikides C, Gaertner L, Luke MA, O’Mara EM, Gebauer JE. A three-tier hierarchy of self-potency: Individual self, relational self, collective self. Advances in experimental social psychology. 2013;48(1):235–95.

[9] Diener E, Diener M. Cross-Cultural Correlates of Life Satisfaction and Self-Esteem. J Pers Soc Psychol. 1995;68:653–63. 7738768 10.1037//0022-3514.68.4.653

[10] Seligman MEP, Csikszentmihalyi M. Positive psychology: An introduction. American Psychologist; American Psychologist. 2000;55(1):5 11392865 10.1037//0003-066x.55.1.5

[11] Diener E, Oishi S, Lucas RE. National accounts of subjective well-being. American Psychologist. 2015;70(3):234–42. doi: 10.1037/a0038899 25844649 10.1037/a0038899

[12] Diener E, Suh E, Lucas R, Smith H. Subjective well-being: Three decades of progress. Psychological bulletin. 1999;125(2):276–302.

[13] Robins RW, Hendin HM, Trzesniewski KH. Measuring global self-esteem: Construct validation of a single-item measure and the Rosenberg Self-Esteem Scale. Personality and Social Psychology Bulletin. 2001;27:151–61.

[14] Steger MF, Frazier P, Oishi S, Kaler M. The meaning in life questionnaire: Assessing the presence of and search for meaning in life. Journal of Counseling Psychology. 2006;53(1):80.

[15] Ryan RM, Frederick C. On energy, personality, and health: Subjective vitality as a dynamic reflection of well‐being. Journal of personality. 1997;65(3):529–65. 9327588 10.1111/j.1467-6494.1997.tb00326.x

[16] Tajfel H. Social psychology of intergroup relations. Annu Rev Psychol. 1982;33:1–39.

[17] Brewer MB, Gardner W. Who is this" We"? Levels of collective identity and self representations. J Pers Soc Psychol. 1996;71(1):83.

[18] Sedikides C, Brewer MB. Individual Self, Relational Self and Collective Self. Philadelphia, PA: Psychology Press; 2001.

[19] Harter S, Waters P, Whitesell NR. Relational self-worth: Differences in perceived worth as a person across interpersonal contexts among adolescents. Child Development. 1998;69:756–66. 9680683

[20] Du H, King RB, Chi P. The development and validation of the Relational Self-esteem Scale. Scandinavian Journal of Psychology. 2012;53:258–64. doi: 10.1111/j.1467-9450.2012.00946.x 22462657 10.1111/j.1467-9450.2012.00946.x

[21] Crocker J, Luhtanen R. Collective self-esteem and ingroup bias. J Pers Soc Psychol. 1990;58:60–7.

[22] Luhtanen R, Crocker J. A collective self-esteem scale: Self-evaluation of one's social identity. Personality and Social Psychology Bulletin. 1992;18:302–18.

[23] Taylor SE, Brown JD. Illusion and well-being: A social psychological perspective on mental health. Psychological Bulletin. 1988;103:193–210. 3283814

[24] Sowislo JF, Orth U. Does low self-esteem predict depression and anxiety? A meta-analysis of longitudinal studies. Psychological bulletin. 2013;139(1):213 doi: 10.1037/a0028931 22730921 10.1037/a0028931

[25] Oishi S, Diener EF, Lucas RE, Suh EM. Cross-cultural variations in predictors of life satisfaction: Perspectives from needs and values. Personality and social psychology bulletin. 1999;25(8):980–90.

[26] Markus HR, Kitayama S. Culture and the self: Implications for cognition, emotion, and motivation. Psychol Rev. 1991;98:224–53.

[27] Crocker J, Luhtanen RK, Blaine B, Broadnax S. Collective self-esteem and psychological well-being among White, Black, and Asian college students. Personality and Social Psychology Bulletin. 1994;20:503–13.

[28] Tajfel H. I6. Instrumentality, identity and social comparisons. Social identity and intergroup relations. 2010:483.

[29] Bettencourt BA, Dorr N. Collective self-esteem as a mediator of the relationship between allocentrism and subjective well-being. Personality and Social Psychology Bulletin. 1997;23(9):955–64. 10.1177/0146167297239005 29506448

[30] Zhang L. Prediction of Chinese life satisfaction: Contribution of collective self-esteem. International Journal of Psychology. 2005;40:189–200.

[31] Simsek OF. Structural Relations of Personal and Collective Self-Esteem to Subjective Well-Being: Attachment as Moderator. Social Indicators Research. 2013:1–18.

[32] Brewer MB, Chen Y-R. Where (who) are collectives in collectivism? Toward conceptual clarification of individualism and collectivism. Psychol Rev. 2007;114(1):133 doi: 10.1037/0033-295X.114.1.133 17227184 10.1037/0033-295X.114.1.133

[33] Baumeister RF, Leary MR. The need to belong: desire for interpersonal attachments as a fundamental human motivation. Psychological Bulletin. 1995;117(3):497 7777651

[34] Diener E, Seligman ME. Very happy people. Psychological science. 2002;13(1):81–4. doi: 10.1111/1467-9280.00415 11894851 10.1111/1467-9280.00415

[35] King RB. Sense of relatedness boosts engagement, achievement, and well-being: A latent growth model study. Contemporary Educational Psychology. 2015;42:26–38.

[36] Madigan S, Atkinson L, Laurin K, Benoit D. Attachment and internalizing behavior in early childhood: A meta-analysis. Developmental Psychology. 2013;49(4):672–89. doi: 10.1037/a0028793 22686171 10.1037/a0028793

[37] Pallini S, Baiocco R, Schneider BH, Madigan S, Atkinson L. Early child–parent attachment and peer relations: A meta-analysis of recent research. Journal of Family Psychology. 2014;28(1):118–23. doi: 10.1037/a0035736 24512287 10.1037/a0035736

[38] Kwan VSY, Bond MH, Singelis TM. Pancultural explanations for life satisfaction: Adding relationship harmony to self-esteem. J Pers Soc Psychol. 1997;73:1038–51. 9364759 10.1037//0022-3514.73.5.1038

[39] Du H, Li X, Chi P, Zhao J, Zhao G. Relational self-esteem, psychological well-being, and social support in children affected by HIV. Journal of Health Psychology. 2014 doi: 10.1177/1359105313517276 24423572 10.1177/1359105313517276

[40] Du H, Jonas E, Klackl J, Agroskin D, Hui EKP, Ma L. Cultural influences on terror management: Independent and interdependent self-esteem as anxiety buffers. Journal of Experimental Social Psychology. 2013;49:1002–11.

[41] Cheng C, Jose PE, Sheldon KM, Singelis TM, Cheung MW, Tiliouine H, et al Sociocultural differences in self-construal and subjective well-being: A test of four cultural models. Journal of Cross-Cultural Psychology. 2011;42(5):832–55.

[42] O’brien RM. A caution regarding rules of thumb for variance inflation factors. Quality & Quantity. 2007;41(5):673–90.

[43] Diener ED, Emmons RA, Larsen RJ, Griffin S. The satisfaction with life scale. Journal of Personality Assessment. 1985;49:71–5. doi: 10.1207/s15327752jpa4901_13 16367493 10.1207/s15327752jpa4901_13

[44] Watson D, Clark L, Tellegen A. Development and validation of brief measures of positive and negative affect: The PANAS scales. J Pers Soc Psychol. 1988;54(6):1063–70. 3397865 10.1037//0022-3514.54.6.1063

[45] Du H, Li X, Chi P, Zhao J, Zhao G. Psychometric properties of the Relational Self-Esteem Scale in a community-based sample in China. European Journal of Psychological Assessment. 2016 doi: 10.1027/1015-5759/a000320

[46] Du H, King RB. Placing hope in self and others: Exploring the relationships among self-construals, locus of hope, and adjustment. Personality and Individual Differences. 2013;54:332–7.

[47] MacKinnon DP, Krull JL, Lockwood CM. Equivalence of the mediation, confounding and suppression effect. Prevention Science. 2000;1(4):173–81. 11523746 10.1023/a:1026595011371 PMC2819361

[48] Nesdale D, Mak AS. Ethnic identification, self-esteem and immigrant psychological health. International Journal of Intercultural Relations. 2003;27(1):23–40.

[49] Greenaway KH, Haslam SA, Cruwys T, Branscombe NR, Ysseldyk R, Heldreth C. From “we” to “me”: Group identification enhances perceived personal control with consequences for health and well-being. J Pers Soc Psychol. 2015. 10.1037/pspi0000019 25938701

[50] Lyubomirsky S, Lepper HS. A measure of subjective happiness: Preliminary reliability and construct validation. Social Indicators Research. 1999;46(2):137–55.

[51] Triandis HC, Gelfand MJ. Converging measurement of horizontal and vertical individualism and collectivism. J Pers Soc Psychol. 1998;74:118–27.

[52] Finkel SE. Causal analysis with panel data Thousand Oaks, CA: Sage Publications; 1995.

[53] Du H, Bernardo ABI, Yeung SS. Locus-of-hope and life satisfaction: The mediating roles of personal self-esteem and relational self-esteem. Personality and Individual Differences. 2015;83:228–33. doi: 10.1016/j.paid.2015.04.026

[54] Cai H, Wu Q, Brown JD. Is self-esteem a universal need? Evidence from The People's Republic of China. Asian Journal of Social Psychology. 2009;12(2):104–20.

